# Single Case Experimental Design: A New Approach for Non-invasive Brain Stimulation Research?

**DOI:** 10.3389/fnrgo.2021.678579

**Published:** 2021-05-31

**Authors:** Joshua W. Pate, Alana B. McCambridge

**Affiliations:** Graduate School of Health, Discipline of Physiotherapy, University of Technology Sydney, Sydney, NSW, Australia

**Keywords:** single case experimental design, non-invasive brain stimulation, repeated transcranial magnetic stimulation, transcranial direct current stimulation, patient-tailored

## Introduction

Due to the large proportion of people living with chronic neurological impairments, non-invasive brain stimulation (NIBS) was developed as a potential adjuvant to enhance neurological rehabilitation. NIBS techniques such as transcranial direct current stimulation (tDCS) and repetitive transcranial magnetic stimulation (rTMS) have been proposed to temporarily modulate neural excitability in a given direction based on the type of stimulation used. For example, seminal studies in tDCS reported that anodal tDCS over the motor cortex (M1) increased corticomotor excitability, whereas M1 cathodal tDCS decreased corticomotor excitability, for up to an hour (Nitsche and Paulus, 2001; Nitsche et al., 2003). The polarity dependent changes in corticomotor excitability were suggested to represent up-regulation or down-regulation of neural plasticity (Nitsche and Paulus, 2001; Nitsche et al., 2003) and depend on several stimulation parameters (Woods et al., 2016). When delivered before or during rehabilitation NIBS may increase the beneficial neuroplastic effects of rehabilitation alone.

Subsequently, the field of NIBS grew rapidly. Many researchers applied tDCS or rTMS to a wide range of clinical populations and explored many variations in experimental parameters. Most commonly, researchers opted to use a randomized controlled trial (RCT) study design, allocating participants to either; real vs. sham stimulation, facilitatory vs. inhibitory stimulation (e.g., anodal vs. cathodal tDCS), stimulation alone vs. stimulation with therapy, or some combination of the above in a multi-arm trial. In addition, NIBS trials have typically used functional and/or neurophysiological outcomes (such as motor learning tasks or measures of corticomotor excitability after M1 stimulation) to determine the after-effects of stimulation, often followed up at multiple time-points to determine the duration of effects. The ease and reported effectiveness of sham stimulation (e.g., fade in-fade out protocol for tDCS, or sham coil rTMS) (Gandiga et al., 2006; Mennemeier et al., 2009) allows robust methodological study designs such as double-blinded cross-over RCTs frequently used in NIBS research.

However, despite the rapid rise of NIBS research over the past 20 years, current evidence has largely not supported translation into clinical practice, with only rTMS as a treatment for drug-resistant depression adopted clinically so far (O'Connell et al., 2018; Elsner et al., 2020). One of the major criticisms of NIBS that likely acts as a barrier to translation is the substantial between-subject variability observed in response to stimulation (López-Alonso et al., 2014; Wiethoff et al., 2014; McCambridge et al., 2015). With some causal studies of healthy participants showing that only half of participants respond to NIBS as expected (López-Alonso et al., 2014; Wiethoff et al., 2014). Therefore, when investigating group-level changes in outcomes the results are unlikely to show statistical or clinically meaningful differences and may mask the positive benefits experienced by some participants. Yet, it is not surprising that substantial between-subject variability exists given the lack of precision in current NIBS studies that typically adopt a “one-size-fits-all” approach to delivery of the intervention.

**Figure 1:**
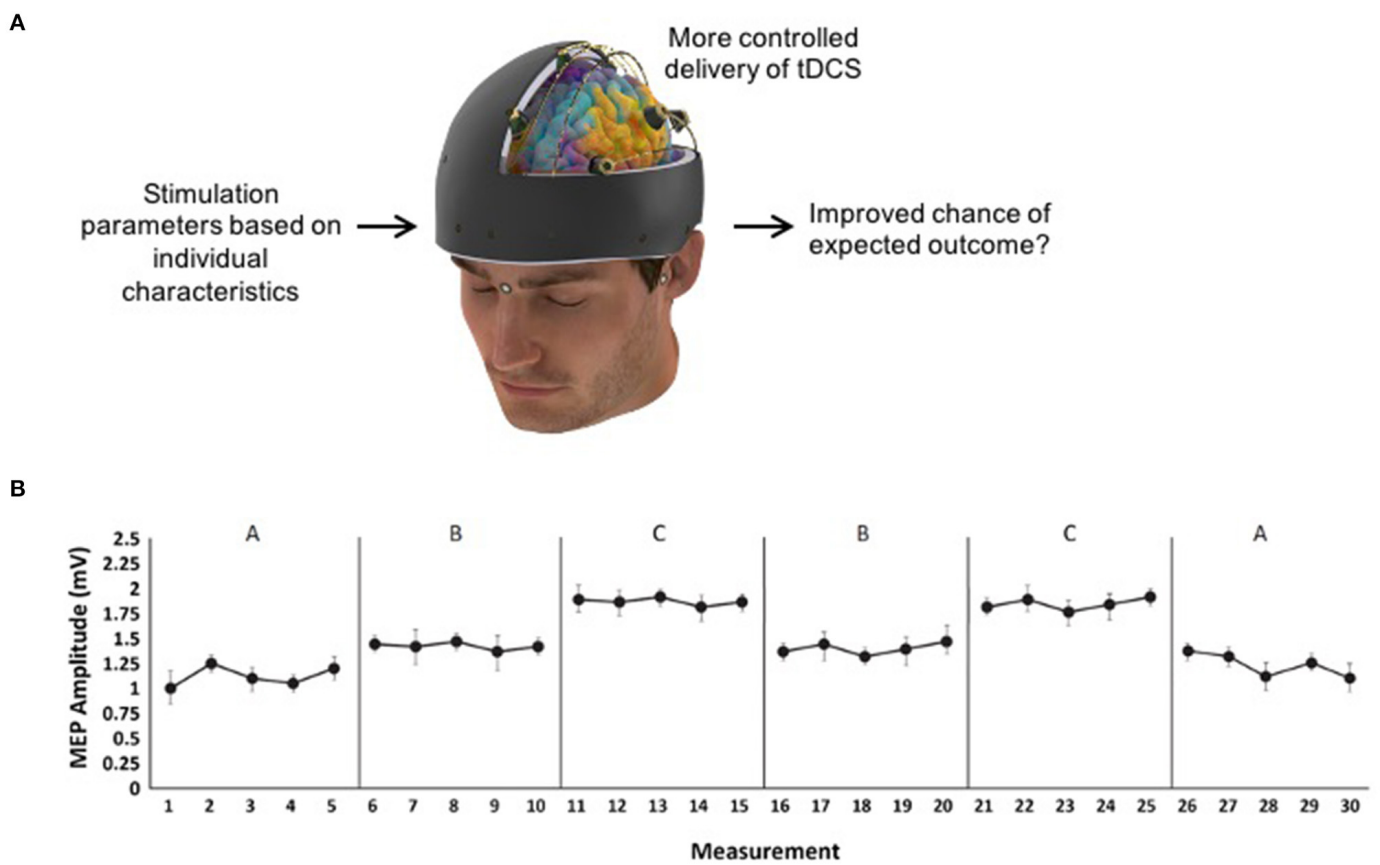
**(A)** Example of individualized transcranial direct current stimulation (tDCS) that could be tested using SCED methodology. **(B)** Hypothetical data for an A-B-C-B-C-A design where five measurements are taken in each of the six phases so that three phase changes between B and C can be reported on (e.g., B → C, C → B, and B → C again).

In the literature, several determinants of NIBS are known to influence stimulation response, both interventional (e.g., type and location of stimulation) and biological factors (e.g., skull thickness, brain morphology and neurochemistry) (Filmer et al., 2019; Hordacre et al., 2021) that should be taken into consideration when delivering NIBS. Researchers have discussed the need for individualized or tailored stimulation protocols, using known determinants of NIBS to guide stimulation protocols to help address wide-scale variability in responses (Di Pino et al., 2014; McCambridge et al., 2018; Hordacre et al., 2021). For example in tDCS research, individualized stimulation could tailor the stimulation protocol based on clinical characteristics of the patient (e.g., lesion location, structural integrity of neural pathways) (Di Pino et al., 2014) or individually modeled electric fields (Antonenko et al., 2019) or a combination of many factors including methodological (e.g., dosage and biological factors) (Hordacre et al., 2021) (Figure 1). Patient-tailored brain stimulation would be a sensible approach, though the feasibility of conducting a large-scale RCT with highly-precise individualized stimulation protocols for each participant would be difficult, particularly in a clinical population. Therefore, in this opinion article, we consider if an alternate methodological approach to investigating the effectiveness of NIBS in lab-based or clinical settings would be of interest to the field.

## Single-Case Experimental Designs

To our knowledge, a study design that has not yet been employed in NIBS research is a single case experimental design (SCED). SCEDs are clinical trials of an individual (or several individuals each studied as a single case), in contrast to trials of groups of participants (Tate and Perdices, 2019). They have also been called single-system designs, single-subject designs, single-case research designs, single participant designs, interrupted time-series designs, and N-of-1 trials. As with many other study designs, the results of SCEDs can be included in systematic reviews (Tanious and Onghena, 2020). To establish a causal relationship between a target behavior and an intervention, SCEDs have four essential elements: (1) the participant is their own control, (2) a priori methods are used, (3) the target behavior is repeatedly measured before, during and after an intervention, and (4) the intervention is systematically manipulated (Tate and Perdices, 2019). Inferences about the intervention are then drawn from the repeated demonstration of the intervention effect on the outcome. Table 1 shows the four classifications of SCED designs: (1) withdrawal/reversal designs, (2) multiple-baselines designs, (3) alternating-treatments designs, and (4) changing-criterion designs. A range of modifications and combinations of these four approaches have been used to date (Shadish and Sullivan, 2011), with some designs better suited to particular type of research questions (see Table 1). One key appeal of a SCED is the ability to individualize an intervention to a given participant, as would be done in many clinical practice settings. For health research, SCEDs are considered Level 1 evidence (OCEBM Levels of Evidence Working Group, 2011) and the methodological quality of SCEDs can be assessed and guided by the recently developed Single-Case Reporting guideline In BEhavioural interventions (SCRIBE) 2016 checklist (Tate et al., 2016a,b).

**Table 1:** Classification of SCED designs adapted from Chapter 1 of Tate and Perdices (2019).

**Classification**	**Research question**	**NIBS example research question**	**When would you choose this approach?**	**Designs**
(1) Withdrawal/reversal designs	What is the effect of systematically introducing and withdrawing the intervention on the target behavior?	Does individualized motor cortex anodal tDCS compared to sham tDCS facilitate corticomotor excitability?	If intervention effects are to be evaluated relative to baseline performance	• A-B-A-B • Multiple-treatment (e.g., A-B-C-B-C-A)
(2) Multiple-baselines designs	What is the effect of an intervention when applied simultaneously, but in a staggered sequence, to different participants, or alternatively, different target behaviors or different settings for the same individual?	Does cognitive training with tDCS whether delivered at home or in clinic improve cognition in people with multiple sclerosis?	If (1) the intervention is likely to produce permanent changes, or (2) it is unethical/impractical to return to baseline conditions	• Across participants • Across behaviors • Across settings
(3) Alternating-treatments designs	What is the effectiveness of two or more interventions (one of which can be a “no-treatment” condition) in the same participant?	Is ipsilesional facilitatory stimulation or contralesional inhibitory stimulation of the motor cortex most effective at increasing paretic arm function after stroke?	If two or more interventions can be provided to the same participant in rapid alternation	• Comparison phase only • Comparison phase with initial baseline • Comparison phase with “best treatment” phase
(4) Changing-criterion designs	How effective is an intervention at gradually inducing therapeutic change in the target behavior?	Is NIBS to the pre-frontal cortex able to gradually reduce food cravings in Bulimia nervosa?	If (1) the intervention is not withdrawn, (2) the same intervention is applied across all the subphases, and (3) the level of target behavior performance in each intervention subphase can become the “baseline” against which performance in the next intervention subphase is compared.	• Standard • Range-bound • Distributed criterion

## Example Protocol for Non-Invasive Brain Stimulation

### Planning

To further elaborate on SCEDs and the potential utility for NIBS research, we propose an example SCED protocol to answer the research question “Is motor cortex anodal tDCS more effective than sham tDCS at increasing corticomotor excitability in chronic stroke participants?” To answer this question, a withdrawal multiple treatment design is appropriate (see Table 1). In this example, an A-B-C-B-C-A design could be used where A = baseline or follow-up with no stimulation, B = sham tDCS, and C = real tDCS. This design provides three opportunities to examine the experimental effect between B and C. To establish the absolute effectiveness of B or C compared to baseline, the design would require at least three phase changes involving A and C, and three involving A and B. This is a very cumbersome design [e.g., A-B-C-B-C-A-B-A-C-A (Tate and Perdices, 2019)]. One ethical and practical consideration here is whether the design should end on an active intervention phase so that the study ends on a potentially beneficial treatment for the patient. The appropriate duration of each phase is dependent on the “wash-out” period needed after stimulation, estimated to be 1–1.5 h for anodal tDCS (Nitsche and Paulus, 2001) thereby minimizing possible confounding “alternation effects” (Tate and Perdices, 2019). Within each phase, at least five measurements of the outcome measure should be taken (see Figure 1B for hypothetical data) to account for possible variability (Kratochwill et al., 2013). In this example, 10 TMS-pulses could be delivered every 4–5 s at each measurement to provide a total of five average MEP amplitudes per phase.

Randomization and blinding are important for the internal validity of a SCED. Randomization can be achieved in some SCED classifications by randomizing the phases, or randomizing the onset in a multiple-baseline study. In our A-B-C-B-C-A example, the sequence of phases is not randomized. However, blinding could be achieved by concealing the stimulation type (real, sham) to the participants and the outcome assessors and not revealing the order of phases or having a separate researcher administer the intervention that is not involved in data collection or analysis. For example, sham tDCS protocols set up the electrodes the same as in real stimulation except the current is ramped up to provide sensory stimulation on the scalp and then ramped down again to no stimulation. Some tDCS machines can also be pre-programmed to do the “fade in-fade out” protocol without the experimenter's knowledge or use a separate experimenter deliver the stimulation. In addition, blinding during the analysis could be achieved by not disclosing the phase (baseline, real, sham, follow up) to the statistician analyzing the data until all analyses are complete.

### Considerations

In terms of reporting on a SCED study, it is important to thoroughly report the selection criteria, participant characteristics, setting, outcome measures, equipment, intervention details, and the procedural fidelity of the intervention. A thorough description is required for replication and to maximize the external validity of the study, for example, researchers may want to repeat the trial on three or more participants that represent the clinical population (i.e., young and old, male and female, different settings etc.).

Detailed descriptions of all phases of the intervention are required (i.e., baseline, real, sham) in SCED studies. If the study uses patient-tailored brain stimulation then a comprehensive description should be given about how stimulation is individualized (i.e., based on what parameters) so that replication of the intervention application and how it was individualized could be achieved in subsequent trials. Reporting how and when the intervention was delivered as well as the intervention dosage (e.g., current strength, duration of stimulation, electrode size and placement etc.) is of particular importance. Similar detail should be given to describing the sham intervention, for example the current strength and duration of the tDCS fade in-fade out protocol (Ambrus et al., 2012).

### Analysis

To begin planning a SCED data analysis, it is important to first determine if changes in level, trend, and/or variability of the outcome measure are indicative of a treatment effect. For example, if the outcome measure was corticomotor excitability assessed with TMS–induced motor evoked potentials (MEP), then a change in the level of corticomotor excitability (i.e., amplitude of the MEPs) with real NIBS compared to sham and baseline would indicate stimulation was effective. Similarly, if the outcome was a motor learning task you may hypothesize a change in the trend (i.e., rate of improvement) with real NIBS would indicate that the treatment was effective. Visual and statistical analysis supplement each other in SCED studies and need to be utilized in conjunction (Tate and Perdices, 2019), though controversy about analysis procedures exist.

The approach selected for visual and statistical analysis is essential to consider. Kratochwill et al. (2013) includes four steps in visually evaluating the phases of a SCED study which can be summarized as: (1) “Is the data stable at baseline?” (2) “Are there any trends or variability?” (3) “How long until there is an effect?” and (4) “Did the control work?” Various techniques exist to visually analyse data in a systematic and objective manner, including descriptive statistics, level changes, split-middle trend lines, variability and evaluating trends in each phase (Parsonson et al., 1992; Lane and Gast, 2014; Barton et al., 2018). For statistical analysis methods, detailed guides are available (Manolov and Solanas Pérez, 2018) because a range of issues may influence the validity of statistical tests (Parker et al., 2011; Velicer and Molenaar, 2013; Harrington and Velicer, 2015). Statistical analyses are valuable (1) when variability appears large upon visual inspection, (2) when effects of interventions are not yet well-understood, (3) when small but important changes in target behaviors cannot be detected by visual analysis, or (4) to enhance replication studies (Kazdin and Tuma, 1982).

## Advantages and Challenges

SCEDs are a useful study design to further explore NIBS, particularly for patient-tailored NIBS protocols. Similar to a RCT, SCEDs are considered Level 1 evidence for health interventions (OCEBM Levels of Evidence Working Group, 2011). One advantage of a SCED over a large-scale RCT is that it is more cost effective, because it requires fewer participants and resources. Not requiring large sample sizes to achieve statistical power may be of particular importance for low-incidence patient populations that often suffer from being under-powered. In addition, because SCEDs use an individual as their own control, outcome measures can be interpreted as absolute values relative to the participant's baseline. Interpretation of absolute values would avoid non-validated normalization procedures used in some neurophysiological research (e.g., pre-post, pre-post/post, pre-post/pre+post). Although tailored-interventions can be used in RCTs, SCEDs are also suitable for patient-tailored treatments that may involve clinical judgements that are commonplace in clinical practice. In a SCED study, individual modifications to the protocol can be extensively explained for each participant so that replication is possible. Furthermore, exploring patient-tailored NIBS with a SCED design can also inform how a tailored intervention could be further tested in a large-scale RCT.

However, there are some important challenges that should be considered. A current challenge to SCED research is the generalizability of findings, particularly for very heterogenous patient populations (e.g., stroke, dystonia). Replication is therefore required, though guidance on how many participants are needed for acceptable replication is lacking. SCED's can also be burdensome to participants because they require extensive data collection. Another challenge is that SCED's are vulnerable to plausible rival hypotheses that may explain the outcomes such as, maturation, regression to the mean and external factors (Caneiro et al., 2019). Further, there are currently no agreed upon statistical analysis procedures. Criticism of the subjective nature of visual inspection, and hence acceptability of SCEDs as Level 1 evidence by the field, could also be a barrier to translation.

## Summary

In summary, the fundamental aspects of NIBS appear to be suitable for studies using SCED methodology. This approach may be a useful avenue to further investigate inter-individual variability and more advanced individualized stimulation protocols. In addition, due to the relative ease and safety of modulating the independent variable in NIBS research, NIBS may also be a candidate field to improve and develop SCED methodology.

## Author Contributions

AM: conception of the idea. AM and JP: manuscript preparation and approval of final paper. Both authors contributed to the article and approved the submitted version.

## Conflict of Interest

The authors declare that the research was conducted in the absence of any commercial or financial relationships that could be construed as a potential conflict of interest.
